# Recreational off-highway vehicle crashes resulting in victims being treated at a regional trauma center: mechanisms and contributing factors

**DOI:** 10.1186/s40621-020-00251-4

**Published:** 2020-06-12

**Authors:** Charles A. Jennissen, Meaghan T. Reaney, Gerene M. Denning

**Affiliations:** 1 Department of Emergency Medicine, Roy J. and Lucille A. Carver College of Medicine, University of Iowa, Iowa City, 52242 USA; 2 Department of Pediatrics, Roy J. and Lucille A. Carver College of Medicine, University of Iowa, Iowa City, 52242 USA

**Keywords:** Recreational off-road vehicles, Utility task vehicles, Side-by-sides, Rollover, Safety belt, Harness, Helmet, Youth, Adolescent

## Abstract

**Background:**

Recreational off-highway vehicles (ROVs) have become increasingly popular in recent years; however, crash epidemiology is not well described. ROVs travel at least 30 mph, and unlike all-terrain vehicles, have a rollover protective structure (ROPS) and seat belts or a harness system for occupants. This study’s objective was to evaluate the demographics, mechanisms, injuries, and associated risk factors of ROV crashes.

**Methods:**

A retrospective chart review was performed for patients of all ages with ROV-related injuries presenting to a Level 1 trauma center from 2004 to 2017. Cases were identified by ICD-9/10 codes and narrative searches. Person- and crash-related variables were examined in relation to injury outcomes including body area injured, injury severity score, and disposition (e.g. hospitalization, intensive care unit admission). Descriptive, bivariate (chi-square, Fishers exact test), and linear regression analyses were performed.

**Results:**

Seventy-two patients with ROV-related injuries were identified. The number of injured patients increased over the study period (*p* < 0.01). Patients were 49% youth < 16 years old, 63% males, and 99% Caucasian. Half of the injured (51%) were passengers, with a higher proportion of youth being passengers (70%) as compared to adults (35%) (*p* < 0.01). Nearly one-third (30%) of crash victims < 16 years old were ROV drivers. Twenty-nine percent of all crashes occurred on roadways. Almost 40% of injured adults crashed at night, while all youth were injured during the day (*p* < 0.01). The primary crash mechanism was a rollover (67%). Only one patient was documented as being helmeted, and approximately one-fourth (24%) sustained head injuries and/or loss of consciousness. Other documented injuries included those to the face (20%), chest (22%), abdomen (11%), extremities (58%), and skin (51%). Over 90% of narratives were consistent with victims being unrestrained. Nearly three-fourths (74%) of victims were hospitalized and 26% required ICU care, one-half (53%) of these being children.

**Conclusions:**

Although ROVs have ROPs, lack of helmet and safety belt use are reducing their benefit. Youth are a large proportion of those injured in ROV crashes, often while driving despite vehicle operation recommended only for those ≥16 years old. Increased public education is needed regarding proper safety measures while operating and riding ROVs.

## Background

Recreational off-highway vehicles (ROVs) are off-road vehicles whose popularity has soared in recent years (U.S. Consumer Product Safety Commission, 2016). Since 2004, world-wide sales of ROVs have continually risen and reached 530,000 vehicles in 2018 (Polaris Industries, n.d.-a). Sales have surpassed that of all-terrain vehicles (ATVs) since 2015 (Polaris Industries, n.d.-a). Consequently, ROV-related deaths and injuries have become an emerging safety issue, particularly among children (Linnaus et al., 2017; Richardson et al., 2018).

ROV types include sport models primarily for recreation, and multipurpose vehicles for both recreational and occupational use. All ROVs have maximum speeds of at least 30 miles per hour (mph), and most can travel at highway speeds (Wilson, 2015). A related off-road vehicle is the utility task vehicle (UTV). ROVs are often referred to as UTVs, but the latter have maximum speeds of < 25 mph (Wilson, 2015).

ROVs are designed solely for off-road use having low pressure tires with knobby treads, and a relatively narrow track and high clearance, which increases their risk of rollover. They also have a steering wheel for directional control, foot pedals for acceleration and braking, and are designed to carry more than one passenger (unlike most ATVs) with bench or bucket seating. ROVs have a rollover protective structure (ROPS) with seat belts or harness restraints for each seating position. In addition, in contrast to most ATVs that have all wheels traveling at the same speed due to a solid rear axle or locked differential, the differential in many ROVs may be selectively unlocked. This allows the outer wheels to rotate faster than the inner wheels when traveling around a corner, similar to a roadway vehicle, and makes the vehicle less likely to tear up turf such as lawns.

Decades of research have provided a wealth of information with regards to ATV crashes and injuries. In contrast, there are very few published studies describing ROV crash and injury epidemiology. Identifying the factors involved in ROV crashes and their associated injuries is vital for developing injury prevention strategies. The objective of this study was to evaluate ROV-related injuries among patients presenting to a Level 1 trauma center.

## Methods

### Patient population

A retrospective chart review was performed for patients of all ages presenting to a Level 1 trauma center with ROV-related injuries from January 1, 2004 to December 31, 2017. Patients were identified in the ED trauma patient database using International Classification of Diseases 9th edition (ICD-9) codes (E821.0-E821.9, E822.2, E823.0-E823.3, E824.0, E824.1, E824.8, E825.0, E825.1-E825.9, E849.1) and 10th edition (ICD-10) codes (V84.5XXA, V86.09XA, V86.55XA, V86.59XA, V86.69XA, V86.79, V86.99XA, V89.1XXA, Y92.79, Y92.71). This study was approved by the authors’ Institutional Review Board.

### Identification of ROV crashes

The ICD codes include crashes of both ATVs and ROVs. To identify the latter, narratives were searched for the terms “ROV” and “UTV,” vehicle makes/models, and mention of any of the following: steering wheel, foot pedals, seat belt, harness, front seat/back seat, more than 4 wheels, rollover protection structure (ROPS), roll cage, or roll bar.

Results from this approach identified 73 cases. Specific keywords found were “UTV” (45 cases), “Gator™” (7 cases), “Rhino™” (1 case), “roll cage” (6 cases), “roll bar” (5 cases), “seatbelt” (7 cases), “steering wheel” (1 case), “back seat” (1 case). One patient was a bystander hit and killed by an ROV when the operator inadvertently accelerated. This case was excluded from analysis.

### Person-related variables

Demographic variables included patient sex, age, race, seating position (operator, passenger), and helmet and/or seatbelt use. Ages were grouped as < 16 (youth) and > 16 years (adult). Other person-related variables were whether the operator tested positive or negative for alcohol use. Drug/medication use was defined as operators testing positive for illicit drugs (e.g. amphetamines) or reporting use of prescription or over the counter (OTC) medications.

For helmet use, the narrative was searched for the word “helmet.” For seatbelts, the narrative was searched using variations on the word “seatbelt” (8 cases). In addition, there were 52 cases where the narrative was consistent with the victim being unrestrained, e.g. the mechanism included being ejected/falling off the vehicle or the victim was found outside/under the vehicle. The narrative was also used to determine whether there was adult supervision of youth operators.

### Crash-related variables

Crash-related variables included the year, the season (coded as Winter (Dec-Feb), Spring (Mar-May), Summer (Jun-Aug), and Fall (Sep-Nov)), and the day of the week grouped as Weekday (Mon-Fri) or Weekend (Sat-Sun). Crash location was coded as on roadways or off-road based on location-related variables. Light conditions were coded as Day, Dawn (30 min before sunrise), Dusk (30 min before sunset), and Night. For bivariate analyses, comparisons were made for Day vs. Night.

The mechanism was coded as a collision with another motor vehicle or an object, or as a non-collision event, either an ejection/fall from the vehicle or a rollover. For bivariate comparisons, crashes were categorized as collision or non-collision events. Narratives were used to determine whether the victim was trapped/pinned under the vehicle. Crashes were also coded as whether they involved multiple victims. Vehicle use was a trauma record coded variable termed work-related. Estimated speeds were extracted from patient notes and grouped as < 20 mph and > 20 mph.

### Outcome variables

Clinical outcomes included the Glasgow Coma Scale (GCS) value at emergency department (ED) presentation. GCS scores were grouped as normal (15) or abnormal (< 15) and by severity; GCS 13–14 (minor), 9–12 (moderate), or severe (< 8). Whether the patient had experienced loss of consciousness (LOC) was also noted.

The maximum abbreviated injury severity (MAIS) score was used to determine whether there was an injury to the head, face, chest, abdomen, extremities, and/or external (skin). MAIS values of 0 were coded as “No” and > 0 as “Yes.” A major trauma was defined as an overall injury severity score (ISS) of > 15. Other injury-related characteristics included whether the patient was admitted to the hospital, admitted to the intensive care unit (ICU), was endotracheally intubated and ventilated, and/or died because of their injuries.

### Data analysis

Descriptive (frequency) and bivariate (chi-square, Fishers Exact test) analyses were performed using IBM SPSS Statistics. Missing data were not included in analysis. Statistical significance for bivariate analysis was defined as a two-sided *p*-value of < 0.05. Linear regression analysis of the data was performed to determine the trendline of the number of ROV victims over time. The β (slope), r^2^ (correlation coefficient) and *p*-value were determined.

## Results

### Person-related characteristics

From 2004 to 2017, 72 patients from 67 crashes presented to the trauma center with injuries as an operator or passenger in an ROV. Patient ages ranged from 4 to 88 years with 49% being youth < 16 years old, and 64% were ≤ 25 years old (Table 1). Almost two-thirds (63%) of patients were male, and 99% were Caucasian. Approximately half (48%) were operators and half (52%) passengers. Less than 10% were belted. Among those adults with alcohol test results (*n* = 23), more than two-fifths (43%) tested positive. Only one patient (a 17-year-old male) was documented as wearing a helmet at the time of the crash. Where supervision of child operators < 16 years old was documented (7/10, 70%), two cases had adult supervision and five were unsupervised.

**Table 1:** Person-related variables for recreational off-highway vehicle (ROV) crash patients presenting to a Level 1 trauma center from 2004 to 2017

	n (Col %)^a^
Group N	72
Sex	
Male	45 (63%)
Female	27 (37%)
Age	
< 16 years	35 (49%)
> 16 years	37 (51%)
Race	
Caucasian	69 (99%)
Other	1 (1%)
Seating	
Operator	34 (48%)
Passenger	36 (51%)
Belted	
Yes	5 (8%)
No	55 (92%)
Alcohol results	
Positive	10 (43%)
Negative	13 (57%)
Drug/medication use	
None documented	48 (67%)
Illicit	2 (3%)
Prescription	15 (21%)
Over the counter	7 (9%)

^a^The sum of n may not equal the total Group N due to missing values

### Crashes

**Fig. 1:**
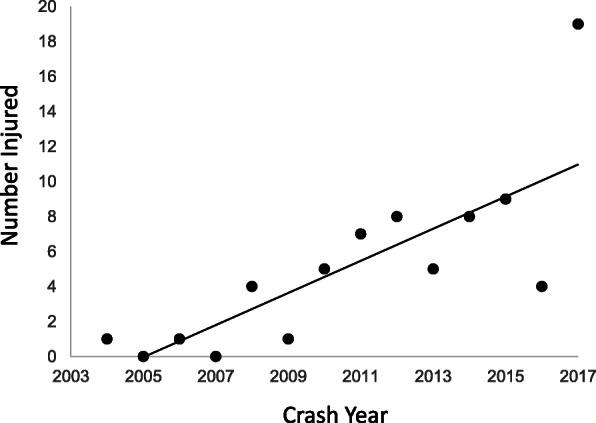
ROV Crash Patients 2004–2017. Graph of the number of ROV crash victims presenting to an academic Level 1 trauma center as a function of crash year. The trendline from linear regression analysis of the data is shown. Results include: β (slope) = 0.95 (indicating an average increase of approximately “1” in the number injured per year), r^2^ (correlation coefficient) = 0.62 (a value defined as a strong correlation), and *p*-value = 0.0008

The number of patients increased over the study period (Fig. 1). The highest proportions of victims were in ROV crashes occurring during the summer months and on weekdays (Table 2). Nearly 30% of crashes were on public roads. Around one in five occurred at night. Two-thirds of victims were injured in a rollover. The second most common mechanism was an ejection/fall from the vehicle. Crash narratives indicated almost two-fifths of victims had at least some part of their body pinned under the ROV, including the ROPS. Four patients were injured while working. Where speed was documented (36/67, 64%), 61% were injured in crashes at speeds of < 20 mph.

**Table 2:** Crash-related variables for recreational off-highway vehicle (ROV) crash patients presenting to a Level 1 trauma center from 2004 to 2017

	n (Col%)^a^
Group N	72
Season	
Winter (Dec-Feb)	4 (6%)
Spring (Mar-May)	22 (31%)
Summer (Jun-Aug)	34 (47%)
Fall (Sep-Nov)	12 (17%)
Day of Week	
Weekday (Mon-Fri)	51 (71%)
Weekend (Sat-Sun)	21 (29%)
Crash Location	
Roadway	12 (29%)
Off-road	30 (71%)
Light	
Day	50 (79%)
Dawn/Dusk	2 (3%)
Night	11 (18%)
Injury Mechanism	
ROV-ROV Collision	1 (1%)
ROV-MV Collision	3 (4%)
ROV-Object Collision	6 (8%)
Ejection/Fall	13 (18%)
Rollover	48 (67%)
Pinned	
Yes	26 (37%)
No	45 (63%)
Work-Related	
Yes	4 (6%)
No	60 (94%)
Speed	
< 20 mph	22 (61%)
> 20 mph	14 (39%)

^a^The sum of n for a variable may not equal the total Group N due to missing data

### Outcomes

Around 10% of patients had an abnormal GCS score < 15 (Table 3). Additionally, six had severe brain injuries (GCS < 8), and 19 suffered a loss of consciousness. Based on MAIS scores, one-fourth of victims suffered a head injury and over half suffered an injury to the extremities and/or externally to the skin. Major trauma (ISS > 15) was observed in 13% of victims. Almost three-quarters of patients were admitted to the hospital, 26% were admitted to the ICU, and 10% were ventilated. About half of those admitted to the ICU (48%) had an ICU length of stay of three or more days. Three patients died (two adults, one youth 6 years old).

**Table 3:** Characteristics of recreational off-highway vehicle (ROV) related crash outcomes among patients presenting to a Level 1 trauma center from 2004 to 2017

	n (Col %)^a^
Group N	72
Glasgow coma scale	
15	63 (89%)
< 15	8 (11%)
Head injury^b^	
Yes	13 (24%)
No	42 (76%)
Face injury^b^	
Yes	11 (20%)
No	44 (80%)
Chest injury^b^	
Yes	12 (22%)
No	43 (78%)
Abdominal injury^b^	
Yes	6 (11%)
No	49 (89%)
Extremity injury^b^	
Yes	32 (58%)
No	23 (42%)
External (skin) injury^b^	
Yes	28 (51%)
No	27 (49%)
Injury severity score	
< 15	63 (87%)
> 15	9 (13%)
Disposition	
Admitted	53 (74%)
Discharged	19 (26%)
Intensive care unit	
Yes	19 (26%)
No	53 (74%)
Ventilated	
Yes	7 (10%)
No	65 (90%)

^a^The sum of n may not equal the total Group N due to missing values

^b^Based on maximum abbreviated injury severity (MAIS) score; 0 = no injury, > 0 = injury

### Males versus females

Comparisons by sex showed females were a higher proportion of youth victims < 16 years old than they were of those ≥16 years (*p* = 0.03) (Table 4). Additionally, relative to males, females were a smaller percentage of patients injured on weekends (*p* = 0.02). No other sex-dependent differences in variables were observed. The patterns and severity of injuries for males and females were also similar (Table 5), with about one-quarter suffering head injuries. Two-thirds of females and almost 80% of males were hospitalized for their injuries. Other results for sex-based comparisons included the observation that 90% of victims testing positive for alcohol were male. Three of the four persons suffering work-related injuries were also male.

**Table 4:** Bivariate analyses of demographics and crashes by sex and by age for victims in recreational off-highway vehicle (ROV) related crashes presenting to a Level 1 trauma center from 2004 to 2017

	Sex			Age		
	Male n (Col %)^a^	Female n (Col %)^a^	*p* value	< 16 Years n (Col %)^a^	> 16 Years n (Col %)^a^	*p* value
Group N	45	27		35	37	
Age						
< 16 years	17 (38%)	18 (67%)	0.03			
> 16 years	28 (62%)	9 (33%)				
Seating						
Operator	24 (55%)	10 (38%)	0.22	10 (30%)	24 (65%)	0.005
Passenger	20 (45%)	16 (62%)		23 (70%)	13 (35%)	
Belted						
Yes	1 (3%)	4 (17%)	0.15	4 (13%)	1 (3%)	0.36
No	35 (97%)	20 (83%)		27 (87%)	28 (97%)	
Season						
Winter (Dec-Feb)	2 (4%)	2 (7%)	0.15	0 (0%)	4 (11%)	0.19
Spring (Mar-May)	13 (29%)	9 (33%)		11 (31%)	11 (30%)	
Summer (Jun-Aug)	19 (42%)	15 (56%)		19 (54%)	15 (41%)	
Fall (Sep-Nov)	11 (24%)	1 (4%)		5 (14%)	7 (19%)	
Day of Week						
Weekday (Mon-Fri)	27 (60%)	24 (89%)	0.02	28 (80%)	23 (62%)	0.12
Weekend (Sat-Sun)	18 (40%)	3 (11%)		7 (20%)	14 (38%)	
Location						
Roadway	7 (28%)	5 (29%)	1.0	6 (26%)	6 (32%)	0.74
Off-Road	18 (72%)	12 (71%)		17 (74%)	13 (68%)	
Light						
Day	29 (81%)	21 (84%)	1.0	33 (100%)	17 (61%)	< 0.001
Night	7 (19%)	4 (16%)		0 (0%)	11 (39%)	
Collision						
Yes	6 (13%)	4 (15%)	1.0	5 (14%)	5 (14%)	1.0
No	39 (87%)	23 (85%)		30 (86%)	32 (86%)	
Pinned						
Yes	27 (60%)	18 (69%)	0.61	12 (34%)	14 (39%)	0.81
No	18 (40%)	8 (31%)		23 (66%)	22 (61%)	
Speed						
< 20 mph	12 (57%)	10 (67%)	0.73	12 (67%)	10 (56%)	0.73
> 20 mph	9 (43%)	5 (33%)		6 (33%)	8 (44%)	

^a^The sum of n for a variable may not equal the total Group N due to missing values

**Table 5:** Bivariate analysis of injuries and outcomes by sex and by age for victims in recreational off-highway vehicle (ROV) related crashes presenting to a Level 1 trauma center from 2004 to 2017

	Sex			Age		
	Male n (Col %)^a^	Female n (Col %)^a^	*p* value	< 16 Years n (Col %)^a^	> 16 Years n (Col %)^a^	*p* value
Group N	45	27		35	37	
GCS						
15	39 (89%)	24 (89%)	1.0	29 (83%)	34 (94%)	0.15
< 15	5 (11%)	3 (11%)		6 (17%)	2 (6%)	
Head injury^b^						
Yes	8 (22%)	5 (26%)	0.75	6 (25%)	7 (23%)	1.0
No	28 (78%)	14 (74%)		18 (75%)	24 (77%)	
Face injury^b^						
Yes	9 (25%)	2 (11%)	0.30	6 (25%)	5 (16%)	0.51
No	27 (75%)	17 (89%)		18 (75%)	26 (84%)	
Chest injury^b^						
Yes	9 (25%)	3 (16%)	0.51	4 (17%)	8 (26%)	0.52
No	27 (75%)	16 (84%)		20 (83%)	23 (74%)	
Abdominal injury^b^						
Yes	4 (11%)	2 (11%)	1.0	2 (8%)	4 (13%)	0.69
No	32 (89%)	17 (89%)		22 (92%)	27 (87%)	
Extremity injury^b^						
Yes	22 (61%)	10 (53%)	0.58	14 (58%)	18 (58%)	1.0
No	14 (39%)	9 (47%)		10 (42%)	13 (42%)	
External (skin) injury^b^						
Yes	18 (50%)	10 (53%)	1.0	12 (50%)	16 (52%)	1.0
No	18 (50%)	9 (47%)		12 (50%)	15 (48%)	
ISS						
< 15	38 (84%)	25 (93%)	0.47	30 (86%)	33 (89%)	0.73
> 15	7 (16%)	2 (7%)		5 (14%)	4 (11%)	
Disposition						
Admitted	35 (78%)	18 (67%)	0.41	23 (66%)	30 (81%)	0.18
Discharged	10 (22%)	9 (33%)		12 (34%)	7 (19%)	
ICU						
Yes	13 (29%)	6 (22%)	0.59	10 (29%)	9 (24%)	0.79
No	32 (71%)	21 (78%)		25 (71%)	28 (76%)	
Ventilated						
Yes	5 (11%)	2 (7%)	0.70	5 (14%)	2 (5%)	0.25
No	40 (89%)	25 (93%)		30 (86%)	35 (95%)	

*Abbreviations GCS* Glasgow Coma Scale, *ICU* Intensive Care Unit, *ISS* Injury Severity Score

^a^The sum of n for a variable may not equal the total Group N due to missing values

^b^Based on maximum abbreviated injury severity (MAIS) score; 0 = no injury, > 0 = injury

### Youth versus adults

A higher percentage of youth were passenger victims compared to those > 16 years (*p* < 0.01) (Table 4). Still, 30% of youth victims were operators of the vehicle. Additionally, compared to 39% of adult patients being injured at night, no victims < 16 years old were in nighttime crashes (*p* < 0.01). Seat belt use by both youth and adults was very low. Similarly to adults, youth were most often injured in the summer and during the week. More than a quarter of youth were injured in a roadway crash, and over 80% of their crashes involved a non-collision event. About a third of youth victims were pinned by the vehicle, and two-thirds of crashes occurred at estimated speeds ≤20 mph.

Injury patterns among youth were similar to adults (Table 5). A quarter of youth suffered injuries to the head and/or face, with one-half or more having extremity or external (skin) injuries. Two-thirds of children were hospitalized. All occupational-related ROV crashes (*n* = 4) and patients testing positive for alcohol (*n* = 10) were adults.

### Other comparisons

Except for the seating differences by age, no differences in crash characteristics and outcomes were observed when comparing operators and passengers. There was a higher percentage of victims testing positive for alcohol on weekends (6/8, 75%), as compared to weekdays (4/15, 27%) (*p* = 0.04). All eight victims with an abnormal GCS were unrestrained, were hospitalized in the ICU, and seven of the eight were mechanically ventilated. Those pinned by the vehicle were more commonly ventilated (5/26, 19%) than those who were not (1/45, 2%) (*p* = 0.02). Pinned victims were also more commonly admitted (23/26, 88%) than those not trapped by the vehicle (29/45, 64%) (*p* < 0.05).

## Discussion

Our study characterized the demographics, crash mechanisms, and clinical outcomes for ROV crash victims presenting to a Level 1 trauma center over a 14-year study period. Overall, we found youth < 16 years old were approximately half (49%) of all victims, rollovers were the major crash mechanism, and personal protective equipment (PPE) was rarely used. Clinical outcomes from these crashes were also relatively severe, with the majority of patients being admitted to the hospital, including 26% to the ICU.

### Recreational off-highway vehicles

Over the last decade, healthcare providers have noticed an increasing number of ROV-related injuries (Linnaus et al., 2017; Richardson et al., 2018). Between January 2003 and August 2016, 942 ROV crashes involving 665 fatalities and 843 injuries were reported by the U.S. Consumer Product Safety Commission (CPSC) (U.S. Consumer Product Safety Commission, 2016). Another study using CPSC and Fatality Analysis Reporting System (FARS) data showed an increase in ROV crash-related deaths on public roadways in the FARS data from two in 2006 to 37 in 2015 (Richardson et al., 2018). A study of newspaper articles from nine Midwestern and Great Plains states from 2009 to 2011 reported 79 crashes with 104 victims (Jennissen et al., 2016). Finally, researchers at a pediatric trauma center studying off-road vehicle crashes from January 2007 through July 2015 identified 42 patients involved in what they termed UTV crashes, most of which were likely ROVs (Linnaus et al., 2017).

### Demographics

Almost two-thirds of patients in our study were males. This proportion is similar to the 2016 CPSC report (68%), the 2018 CPSC/FARS study (71%), and the newspaper article study (70%) (U.S. Consumer Product Safety Commission, 2016; Richardson et al., 2018; Jennissen et al., 2016). In contrast, the pediatric trauma center study reported males comprised only 55% of victims (Linnaus et al., 2017). All of these studies, including ours, found a lower proportion of males than is commonly seen for ATVs, where they are typically > 85% of victims (Denning et al., 2013a; Denning et al., 2013b; Denning et al., 2014; Denning & Jennissen, 2016).

ROVs are designed for multiple riders. Consistent with this, we found about half of those injured were passengers. The newspaper article study reported passengers were 37% of victims (Jennissen et al., 2016). In contrast to ROVs, almost all ATVs are designed for an operator only, and previous studies found passengers were about 15–22% of ATV-related injuries and deaths (Jennissen et al., 2016; Denning et al., 2013a; Denning et al., 2013b; Denning et al., 2014; Denning & Jennissen, 2016). These ATV studies found injured females were more commonly passengers than were males. This was also true for ROV crashes in the study using newspaper articles: males, 75% operators, 25% passengers; females, 43% operators, 57% passengers (Jennissen et al., 2016). We saw a similar pattern in our study (Table 4), but the differences did not reach statistical significance.

Children and adolescents appear to be a particularly vulnerable riding population. Nearly one-half (49%) of the ROV crash victims in our study were <  16 years old. This was similar to the newspaper article study showing youth were 44% of ROV crash victims (Jennissen et al., 2016). In both cases, youth were a considerably higher percentage of victims than that typically reported for ATVs (24–29%) (Jennissen et al., 2016; Denning et al., 2014; Denning & Jennissen, 2018). A significantly higher proportion of injured children were passengers (70%) compared to adults (35%) in our study. Still, 30% of child crash victims were drivers of the ROV at the time of their injury. Children < 16 years old were drivers in 21% of crashes in reports collected by the CPSC where the age of the driver was known, and 14% of ROV drivers killed on public roads in the FARS database (Richardson et al., 2018).

Manufacturers warn consumers in the owner’s manuals (Kawasaki Heavy Industries, 2012; Polaris Industries, n.d.-b) and with vehicle decals that adult-size ROVs should not be driven by anyone < 16 years old. Manufacturers also recommend that child passengers be at least 12 years old. They state all riders should be able to sit with their backs against the seat with both feet flat on the floor and grip the passenger hand hold (Kawasaki Heavy Industries L, 2012; Polaris Industries, n.d.-b). Unfortunately, many families do not adhere to these recommendations. Sometimes parents will put children in car seats or booster seats in ROVs, but these products have not been tested for use in these vehicles and manufacturers provide no guidance. Greater efforts are needed to educate consumers on the dangers of ROVs and provide safety information on their proper use.

### Crash mechanism

Rollovers were the primary crash mechanism in our study. Other studies have found the majority of ROV crashes (Jennissen et al., 2016; U.S. Consumer Product Safety Commission, 2014), like ATV crashes (Denning et al., 2014; Unni et al., 2012; Humphries et al., 2006), were rollovers. In addition, the CPSC study determined 68% were lateral rollovers with over one-half occurring during a turn. About 90% of the most severely injured had been in a rollover, and among rollover crashes for which terrain was known, more than half occurred on flat surfaces. The lateral stability and vehicle handling characteristics of ROVs have been a concern (U.S. Consumer Product Safety Commission, 2016; U.S. Consumer Product Safety Commission, 2014), and efforts to improve their safety engineering and design is very much needed.

### Personal protective equipment

A major contributor to severe injury in ROV crashes is riding unrestrained. Only 8% of victims presenting to our trauma center appeared to be restrained, very similar to the 6% of victims documented as being restrained in the newspaper article study (Jennissen et al., 2016). In that same study, nearly three-quarters of those who died had been ejected from the vehicle (Jennissen et al., 2016). In reports using national data, only 14% of ROV occupants were known to be belted, and half to three-quarters of victims who were injured or killed were not wearing a seat belt or harness system (Richardson et al., 2018; U.S. Consumer Product Safety Commission, 2014). The vast majority of these victims were partially or fully ejected from the vehicle. Among pediatric patients with ROV-related injuries, 70% were not restrained (Linnaus et al., 2017).

We found 37% of the victims had been pinned by the vehicle. Other studies have shown about one-half or more of those injured were struck or pinned (Jennissen et al., 2016; U.S. Consumer Product Safety Commission, 2014). Nearly 30% of victims in reports collected by the CPSC were pinned by the vehicle, usually by the ROPS structure (Richardson et al., 2018). ROVs weigh up to a 1000 pounds. They can transmit significant forces to occupants who are ejected from the vehicle and cannot be easily lifted off a pinned victim. One of the most important factors in decreasing ROV-related injuries and deaths is for occupants to always wear the seat belt or harness system for each and every ride, and there should be no passengers who cannot be properly restrained.

Manufacturers and safety experts recommend the use of helmets when riding in ROVs. Only one patient seen at our trauma center was documented as wearing a helmet, and one-fourth of victims suffered a head injury. In a nine-state study of ROV crashes, three (3%) were reported as being helmeted (Jennissen et al., 2016), and < 15% of pediatric victims injured in ROV crashes were wearing helmets, both significantly lower percentages than helmet use on ATVs and dirt bikes (Linnaus et al., 2017). Only 2% of victims in the CPSC database were documented as being helmeted (Richardson et al., 2018).

### Nighttime driving

Despite ROVs being equipped with headlights, two-fifths of adults were injured in nighttime crashes. This is similar to the proportion of all ROV crashes occurring in compromised light conditions in the newspaper article study (Jennissen et al., 2016). Appreciating terrain changes and identifying obstacles is inevitably more difficult at night. Nighttime ROV driving should be done with extreme caution and avoided if possible. Adult alcohol use is also likely to be playing a factor in many adult ROV crashes at night.

### Roadways

Like ATVs, ROVs are designed for off-road use only and manufacturers strongly warn against roadway riding (Specialty Vehicle Institute of America, n.d.; Recreational Off-Highway Vehicle Association, n.d.-a). For both vehicles, the dangers of riding on the road are largely related to their fundamental vehicle design (Jennissen et al., 2016; Denning & Jennissen, 2016; Denning & Jennissen, 2018). Despite the warnings, almost 30% of crashes in our study occurred on public roadways. This proportion is slightly lower than that found in other studies. Specifically, one-half of crashes in the newspaper articles from nine states (Jennissen et al., 2016) and 43% of crashes in the CPSC database (Richardson et al., 2018) were on the road. Interestingly, four-fifths of ROV crashes reported in newspapers from nine states (Jennissen et al., 2016) and 82% of fatal ROV crashes in the FARS (Richardson et al., 2018) occurring on public roads did not involve another motor vehicle. In other words, the vast majority were single vehicle crashes. Similarly, ATV crash studies have shown that over two-thirds of deaths and three-fourths of injuries on public roadways do not involve another motorized vehicle (Denning et al., 2013a; Denning et al., 2013b). Because of their design, off-road vehicles including ROVs can have unpredictable interactions with roadway surfaces and should not be driven on public roads.

### Clinical outcomes

Injuries sustained in ROV crashes are often quite severe. In our study, 74% of patients were hospitalized, including over one-fourth in the ICU, and half of these were youth < 16 years old. A study at a Level 1 pediatric trauma center (2007–2018) identifying 42 ROV crash patients also found high rates of admission to the hospital (79%) and ICU (27%) (Linnaus et al., 2017).

### Limitations

Our study was retrospective, had a relatively limited sample size, and involves a single trauma center, which limits its generalizability to other populations. As potential cases for our study were determined by ICD coding, it is possible some ROV cases were not identified due to miscoding and/or missing narrative cues. In addition, narratives where patients were clearly ejected/fell from the vehicle were interpreted as their being unbelted. However, they could also represent improper belt use. Moreover, the need to identify ROVs and code other variables from narratives may introduce documentation bias. Finally, ED populations do not include all fatalities as those occurring pre-hospital are not included in the trauma database. Despite these limitations, ROV-related crashes and injuries are an emerging safety issue about which little has been published, and our findings contribute to this small body of knowledge.

## Conclusions

Although ROVs have rollover protective structures, lack of adherence to manufacturer safety recommendations including helmet and safety belt use and refraining from driving on public roadways is likely reducing their benefit. Almost all ROVs are designed to be driven by those ≥16 years old, and manufacturers recommend passengers be 12 years or older. Our studies support the hypothesis that youth are a vulnerable ROV riding population. Laws as outlined in the Recreational Off-Highway Vehicle Association’s model legislation (Recreational Off-Highway Vehicle Association, n.d.-b) should be adopted and strictly enforced. Increased public education and anticipatory guidance by healthcare providers on ROV safety are also greatly needed. Given the increasing popularity and prevalence of these vehicles, ROV crashes and injuries should be considered an emerging public health concern and multi-pronged strategies should be developed to prevent these serious injuries.

## Data Availability

Data and materials are available to other parties for research purposes after a data sharing agreement plan is agreed to and signed.

## Abbreviations

AIS: Abbreviated Injury Score
ATV: All-terrain vehicle
ED: Emergency department
GCS: Glasgow Coma Scale
ISS: Injury Severity Score
LOC: Loss of consciousness
LOS: Length of stay
MAIS: Maximum Abbreviated Injury Score
MV: Motor vehicle
ROV: Recreational off-highway vehicle
SPSS: Statistics Package for the Social Sciences
SxS: Side-by-side
UTV: Utility task vehicle

## References

[CR1] Denning G, Harland K, Ellis D, Jennissen C (2013). More fatal all-terrain vehicle crashes occur on the roadway than off: increased risk-taking characterises roadway fatalities. Inj Prev.

[CR2] Denning G, Harland K, Jennissen C (2014). Age-based risk factors for pediatric ATV-related fatalities. Pediatrics.

[CR3] Denning G, Jennissen C (2018). Pediatric and adolescent injury in all-terrain vehicles. Special issue: epidemiology of youth injury in adventure and extreme sports. Res Sports Med.

[CR4] Denning G, Jennissen C, Harland K, Ellis D, Buresh C (2013). All-terrain vehicles (ATVs) on the road: a serious traffic safety and public health concern. Traffic Inj Prev.

[CR5] Denning GM, Jennissen CA (2016). All-terrain vehicle fatalities on paved roads, unpaved roads, and off-road: evidence for informed roadway safety warnings and legislation. Traffic Inj Prev.

[CR6] Humphries RL, Stone CK, Stapczynski JS, Florea S (2006). An assessment of pediatric all-terrain vehicle injuries. Pediatr Emerg Care.

[CR7] Jennissen C, Harland K, Denning G. Characteristics of side-by-side vehicle crashes and related injuries as determined using newspaper reports from nine U.S. States. Safety. 2016;2(2) Available at: http://www.mdpi.com/2313-576X/2/2/10. Accessed 30 Dec 2019. 10.3390/safety2020010 PMC9380433 35979514

[CR8] Kawasaki Heavy Industries. Mule 4010 Trans4x4 Utility Vehicle Owner’s Manual. 2012. Available at: http://www.manualslib.com/manual/803081/Kawasaki-Mule-4010-Trans-4x4.html?page=7#manual Accessed 30 Dec 2019.

[CR9] Linnaus ME, Ragar RL, Garvey EM, Fraser JD (2017). Injuries and outcomes associated with recreational vehicle accidents in pediatric trauma. J Pediatr Surg.

[CR10] Polaris Industries. Polaris 2015-2018 Annual reports. n.d.-a. Available at: http://www.annualreports.com/Company/polaris-industries. Accessed 30 Dec 2019.

[CR11] Polaris Industries. Polaris RZR® 1000 EPS 2019 Owner’s Manual. n.d.-b. Available at: https://www.manualslib.com/manual/1513963/Polaris-Rzr-Xp-1000-Eps-2019.html#product-RZR XP 1000 EPS 2019. Accessed 30 Dec 2019.

[CR12] Recreational Off-Highway Vehicle Association. Position in opposition to on-highway operation of ROVs. n.d.-a. Available at: https://rohva.org/wp-content/uploads/2019/02/ROVOn-HwyPositionPaper.pdf Accessed 30 Dec 2019.

[CR13] Recreational Off-Highway Vehicle Association. Model state recreational off-highway vehicle legislation. n.d.-b. Available at: https://rohva.org/wp-content/uploads/2019/02/ROV_Model_Summary_12-11.pdf Accessed 30 Dec 2019.

[CR14] Richardson RE, McMurry TL, Gepner B, Kerrigan JR (2018). Field data analysis of recreational off-highway vehicle crashes. Traffic Inj Prev.

[CR15] Specialty Vehicle Institute of America. SVIA position in opposition to on-road operation of ATVs. n.d.. Available at: https://svia.org/wp-content/uploads/2017/12/SVIAOnRoadOppositionPosition-2016.pdf Accessed 30 Dec 2019.

[CR16] U.S. Consumer Product Safety Commission (2014). Notice of Proposed Rulemaking. 16 CFR Part 1422. Safety standard for recreational off-highway vehicles (ROVs). Fed Regist.

[CR17] U.S. Consumer Product Safety Commission. Evaluation of Voluntary Standards for Recreational Off-Highway Vehicles (ROVs). 2016. Available at: https://www.cpsc.gov/s3fs-public/RecreationalOffHighwayVehiclesTerminationofRulemaking.pdf Accessed 30 Dec 2019.

[CR18] Unni P, Morrow SE, Shultz B (2012). Analysis of pediatric all-terrain vehicle trauma data in middle Tennessee: implications for injury prevention. J Trauma Acute Care Surg.

[CR19] Wilson L (2015). Side-by-side off-road vehicles: rollover occupant protection—standards and vehicle classification. Winter meeting of the ANB45(1) Rollover subcommittee sponsored by the ANB45 Occupant Protection committee of the Transport Research Board.

